# Composition of the Influence Group in the *q*-Voter Model and Its Impact on the Dynamics of Opinions

**DOI:** 10.3390/e26020132

**Published:** 2024-01-31

**Authors:** Tomasz Weron, Piotr Nyczka, Janusz Szwabiński

**Affiliations:** Department of Applied Mathematics, Wroclaw University of Science and Technology, 50-370 Wroclaw, Poland; tomasz.weron@pwr.edu.pl (T.W.); piotr.nyczka@pwr.edu.pl (P.N.)

**Keywords:** opinion dynamics, *q*-voter model, agent-based modeling

## Abstract

Despite ample research devoted to the non-linear *q*-voter model and its extensions, little or no attention has been paid to the relationship between the composition of the influence group and the resulting dynamics of opinions. In this paper, we investigate two variants of the *q*-voter model with independence. Following the original *q*-voter model, in the first one, among the *q* members of the influence group, each given agent can be selected more than once. In the other variant, the repetitions of agents are explicitly forbidden. The models are analyzed by means of Monte Carlo simulations and via analytical approximations. The impact of repetitions on the dynamics of the model for different parameter ranges is discussed.

## 1. Introduction

According to the Oxford Languages online dictionary, opinion is “a view or judgment formed about something, not necessarily based on fact or knowledge”. In modern societies, due to the ongoing growth of communication technologies and social media platforms, people are constantly exposed to a steady flow of opinions about new technologies, products, or ideas [1]. By processing this flow and interacting with others, individuals may change their own opinions and beliefs [2]. Thus, opinions are an integral part of people’s perception of reality. They shape social behavior and play a significant role in the evolution of societies.

Currently, agent-based models (ABMs) are one of the most popular and efficient tools in opinion dynamics and other social process studies. They can provide a detailed representation of reality, preserving the heterogeneity of individuals and an irregular structure of their mutual relations [3]. In social sciences, ABM is most often understood as a simulation of the behaviors of these individuals, called agents, and interactions between them. The aforementioned structure usually illustrates a network of friendships, contacts, or cooperation, within which agents’ actions take place [4]. From a mathematical point of view, that structure can be represented by a graph, with vertices being the agents and edges—the connections between them [5].

In the case of complex problems such as the dynamics of opinions, simple deterministic models are often found to be insufficient [6]. They are complemented by ABMs, as the latter allow for bridging the gap between microscopic interactions of the agents and emergent phenomena at the macroscopic scale. For many years, ABMs have found use in various fields of social science. For example, in examining the diffusion of innovations, such as new products and market solutions [7,8,9,10,11,12], or in modeling the results of democratic elections and public debates [13,14,15,16,17,18,19,20,21].

Within the field of opinion dynamics, there exists a wide range of different ABMs [22]. However, if we narrow our focus to binary opinions, then the *q*-voter model will come out as one of the most successful ones [23]. Social conformity is the main driving force in this model, and its dynamics are as follows. In each elementary event, we randomly choose a single agent and *q* of its neighbors (hence the name *q*-voter model) within the underlying graph structure. Then, if and only if all *q* neighbors, forming the so-called group of influence, present the same opinion, the agent conforms to it and changes its opinion accordingly.

Although the above requirement of full unanimity may seem too strict, it is strongly supported by the results of Asch’s social experiment [24]. It clearly showed that conformity, i.e., adjusting to group behavior, plays an important role in our decision making and that its impact is severely reduced in the case of disagreement within a group. Consequently, unanimity is not the part of the *q*-voter model that causes our concern. The element that does is the composition of the group of influence. In the original formulation of the model the authors wrote: *“In order to simplify the numerical analysis, and allow for an arbitrary value of q in regular lattices, we consider here the possibility of repetition, i.e., a given neighbor can be selected more than once”* [22]. In the article they only briefly discuss outcomes when repetitions are prohibited, without much detail.

Since then, there has been a lot of research conducted on the *q*-voter model. Some authors followed the original assumption and studied the model with the possibility of repetitions [25,26,27]. Others took an opposite path and considered a variant without them [28,29,30,31,32,33], sometimes justifying it sociologically [34]. There are many papers that do not specify it at all [35,36,37,38]. To the best of our knowledge, there exists only one article that considers differences between these two variants in detail [39]. It does however mostly focus on the so-called threshold *q*-voter model, an extension that incorporates a threshold mechanism [40]. Still, it provides a valuable insight on the differences between the two aforementioned variants. We hope there is room for more.

Here, we consider two different compositions of the influence group, with or without repetitions. We examine their impact in the *q*-voter model with independence [28]. We do so by the means of both computer simulations and analytical approximation methods. As for the latter, the first method is a simple Mean-Field Approximation (MFA) [22]. We are aware that MFA is sufficient only for complete graphs or other dense networks. When a network is sparse, it fails. Despite its limitations, we incorporate it into this work, as it has never been used to describe a variant without repetitions, to the best of our knowledge. Nevertheless, more sophisticated recipes are also needed. Thus, the second method is the Pair Approximation (PA), which works reasonably well, even for sparse networks [29]. Unfortunately, a closed-form solution is possible to obtain only in the variant without repetitions. The repetitive version must be solved numerically [39]. In this paper, we propose yet another solution—a heuristic MFA. This method will provide closed-form solutions for both variants of the *q*-voter model. Moreover, these formulas will be much simpler than in the case of PA.

The rest of the paper is organized as follows. In Section 2, we provide details on the model, its variants and methods used to analyze them. Later, in Section 3 we present and discuss the results. Finally, the conclusions are drawn in Section 4.

## 2. Models and Methods

### 2.1. Simulation Model

Our starting point is the *q*-voter model with independence [28]. We consider a set of *N* agents, each of which is characterized by a binary variable—an opinion, positive or negative, on some given issue, $S_{i}=\pm 1$ for i=1,2,…,N. We investigate the model through Monte Carlo simulations with a random sequential updating scheme. Within a single simulation, the time is measured in so-called Monte Carlo steps (MCS). One MCS consists of *N* elementary events, each divided into the following substeps of length $\Delta t=\frac{1}{N}$:

1. Select a target agent *i* randomly (uniformly from *N* nodes).
2. Draw a random number r∼U(0,1).
3. With probability *p* (that is if r<p), the agent acts independently, i.e., it changes its opinion to the opposite one with probability $f=\frac{1}{2}$, $S_{i}\left(t+\Delta t\right)\overset{f}{\leftarrow }-S_{i}\left(t\right)$.
4. With the complementary probability 1−p (if r>p), a group of influence is constructed:
  - (*repetition* variant) Randomly select *q* neighbors of agent *i*, $j_{1}$, $j_{2}$, ⋯, $j_{q}$, with possibility of repetition.
  - (*no repetition* variant) Randomly select *q* neighbors of agent *i*, $j_{1}$, $j_{2}$, ⋯, $j_{q}$, without the possibility of repetition.
5. If the group of influence is unanimous, $S_{j_{1}}\left(t\right)=S_{j_{2}}\left(t\right)=\cdots =S_{j_{q}}\left(t\right)$, the agent *i* conforms, i.e., $S_{i}\left(t+\Delta t\right)\leftarrow S_{j_{1}}\left(t\right)$. Otherwise, nothing happens.

**Figure 1:**
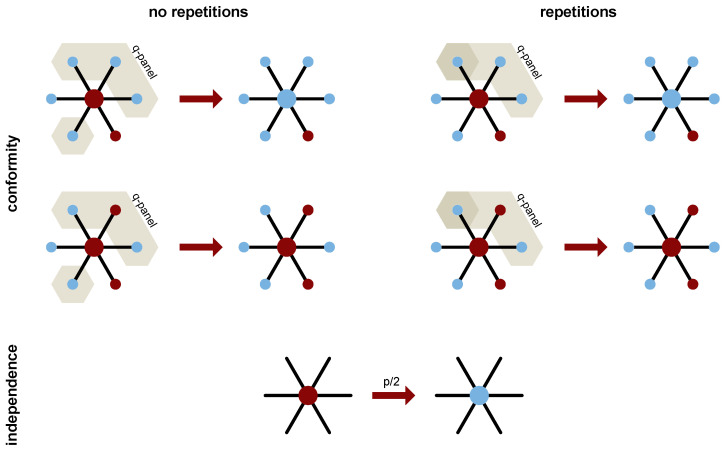
Depiction of the model dynamics in the *no repetition* (**left**) and the *repetition* (**right**) variants. The upper part corresponds to conformist behavior (probability 1−p), while the bottom one corresponds to independence (probability *p*). The big circle portrays the target agent, smaller ones portray its neighbors. Light blue color represents a positive opinion (S=+1), dark red represents a negative one (S=−1), and beige marks a randomly chosen group of influence, with darker beige for repetitive choice. Degree k=6 and size of the influence group q=4 in all the above cases.

As already mentioned above, we consider two variants of the model: the *repetition* and the *no–repetition* one (see Figure 1 for a schematic representation). We decided to examine their behavior and the differences between them on random regular graphs. Other choices for the underlying topology are, of course, possible, but this particular type of complex networks allows us to control the number of neighbors of each agent and to ensure that forming of the influence group will be always possible in the *no–repetition* variant. Thus, those networks seem to be the best choice to implement the group dynamics, as they allow the option to leave out the heterogeneity-induced effects and focus only on the ones related to the differences in the dynamics. It should be noted that the same choice was made in a similar context in Ref. [39].

All methods presented in this work have also been tested on other networks, including square lattices, Watts-Strogatz graphs and scale-free networks. The results turned out to be qualitatively the same as for the random regular graphs.

In order to analyze stationary states, we define a macroscopic measure—the concentration of positive opinions (S=+1):

(1) $$c^{+}=\frac{N^{+}}{N}=\frac{1}{2N}\sum_{i=1}^{N}\left(S_{i}+1\right).$$

We will use it as our default measure throughout this paper. For simplicity, we will just call it “concentration” and omit the superscript + ($c^{+}\to c$).

To clarify, we perform simulations as follows. First, we generate a graph structure with a given degree *k* and assign agents their initial opinions S(0), according to a specified initial concentration $c_{0}$, which can be understood as the probability of an agent having a positive opinion S(0)=+1. Then, we run the simulation until the system reaches its stable state and save final concentration. We repeat such simulation multiple times for a given set of parameters and average the results over these independent runs.

### 2.2. Mean-Field Approximation

Within the mean-field approximation we abstract away from the actual network of connections between the agents and assume that every agent may interact with anybody else [25]. In each elementary step, the number of agents in the state S=1 may increase by 1, decrease by 1 or remain unchanged. The corresponding transition probabilities of the first two events in the repetition variant are qiven by

(2) $$\begin{array}{cc} \mathrm{Pr}\left(c\left(t+\Delta t\right)\leftarrow c\left(t\right)+\Delta_{N}\right) & =\left(1-p\right)\alpha^{+}+p\beta^{+},\\ \mathrm{Pr}\left(c\left(t+\Delta t\right)\leftarrow c\left(t\right)-\Delta_{N}\right) & =\left(1-p\right)\alpha^{-}+p\beta^{-}, \end{array}$$

where

(3) $$\begin{array}{cc} \alpha^{+}=\left(1-c\right)c^{q}, & \;\;\;\;\alpha^{-}=c{\left(1-c\right)}^{q},\\ \beta^{+}=\frac{1}{2}\left(1-c\right), & \;\;\;\;\beta^{-}=\frac{1}{2}c. \end{array}$$

Assuming N→∞, the following dynamical equation may be derived from the above probabilities:

(4) $$\frac{\partial c}{\partial t}=\left(1-p\right)\alpha +p\beta ,$$

where

(5) $$\begin{array}{cc} \alpha & =\alpha^{+}-\alpha^{-},\\ \beta & =\beta^{+}-\beta^{-}. \end{array}$$

In the stationary state, we have $\frac{\partial c}{\partial t}=0$. This leads to the following relationship between the probability of independence *p* and the stationary concentration *c* (hidden in α and β):

(6) $$p=\frac{\alpha }{\alpha -\beta }.$$

The basic mean-field approximation does not distinguish between the variants of the model. However, we can modify it slightly to catch the characteristics of the *no-repetition* dynamics. We retain the assumption about the infinite size of the system (N→∞). Additionally, we introduce a finite degree (and equal for all the agents) *k*. Then, $\alpha^{+}$ and $\alpha^{-}$ take the following forms:

(7) $$\begin{array}{cc} \alpha^{+} & =\left(1-c\right)\prod_{i=0}^{q-1}\max\left[\frac{k\times c-i}{k-i},0\right],\\ \alpha^{-} & =c\prod_{i=0}^{q-1}\max\left[\frac{k\times (1-c)-i}{k-i},0\right], \end{array}$$

while $\beta^{+}$ and $\beta^{-}$ remain unchanged. The rest, including the condition for stationary states (Equation (6)), is the same as in the *repetition* variant. In the remaining part, we call this approach a network aware MFA (naMFA). It should be emphasized here that in the model with repetitions, naMFA reduces to MFA.

### 2.3. Pair Approximation

The pair approximation (PA) is a moment closure method in which the mean-field description of a model is supplemented by an approximate equation for the time evolution of the density of the active links, i.e., the edges in a network joining two agents in different states. PA is one of the possible ways to incorporate the structure of the underlying network into the description of the model. This approximation has been already used for the *q*-voter model with independence [29,39] and turned out to yield precise results for a wide variety of networks. However, regardless of all its advantages, it has a major flaw—it provides a closed-form solution only for the *no repetition* variant. For the *repetition* one, it seems impossible to obtain such a solution [39]. Therefore, we must rely on a numerical one. Although the latter can be very precise, it is not as satisfactory and informative as an analytical formula.

A detailed derivation of PA may be found in Refs. [29,39]. Here, we will only recall the most important results for the *q*-voter model on random regular graphs.

To recall, the degree distribution of a random regular graph is given by $P\left(k^{\prime }\right)=k\delta_{k,k^{\prime }}$, where *k* is simply the degree of each node in the network. Following [39], the model reduces in this case to two closed rate equations for the density of active links ρ(t) and the concentration c(t) of agents in state S=1:

(8) $$\begin{array}{ccc} \frac{d\rho }{dt} & = & 2\sum_{i=⊕,⊖}P_{i}{\langle (k-2l)F(l;k,q,p)\rangle }_{\rho_{i}}\\ \frac{dc}{dt} & = & -\sum_{i=⊕,⊖}S_{i}P_{i}{\langle F(l;k,q,p)\rangle }_{\rho_{i}} \end{array}$$

Here, $S_{⊕}=1$, $S_{⊖}=-1$, $P_{⊕}=c$, $P_{⊖}=1-c$, $\rho_{⊕}=\rho /\left(2c\right)$, $\rho_{⊖}=\rho /\left(2\left(1-c\right)\right)$ and ${\langle \ldots \rangle }_{\rho_{i}}$ is the average calculated over the binomial probability k′lρil(1−ρi)k′−l. The probability that an agent with *l* active links flips its state is

(9) $$F\left(l;k,q,p\right)=\frac{p}{2}+\left(1-p\right)f\left(l;k,q\right),$$

where

(10) f(l;k,q)=k−ql−q/klno-repetition,lkq,repetition.

Moreover, in the no-repetition variant it is understood that f(l;k,q)=0 if k<q.

Due to some cancellations of the combinatorial numbers, the averages ${\langle \ldots \rangle }_{\rho_{i}}$ in Equation (8) in the no-repetition variant lead to relatively simple expressions which are linear in *k*. In this case, the condition dc/dt=0 yields the following relationship between the independence *p* and the stationary concentration *c*:

(11) $$p^{-1}=1+\frac{2^{q-1}{\left(\frac{k-1}{k-2}\right)}^{q}}{q-1}.$$

In the model with repetitions, a closed-form expression does not exist, and one has to resort to numerical solutions. A difficulty in the numerical calculations relates to the fact that the binomial coefficients appearing in the rate equations take huge values for large *k*, while $\rho^{k}$ is very small. To avoid this problem, Vieira et al. [39] made expansions in Equation (8) using the moments of the binomial distribution and the negative moments of the degree distribution.

In this work, we followed a slightly different approach. We took a log-transformed version of Equation (8) and solved it numerically for a given value of *p* (and other parameters of the model). The times taken were long enough to arrive at a stationary solution. The concentration at the last point along the time axis together with the corresponding *p* was stored as the contribution to the p(c) relationship, and the procedure was repeated for a different value of *p*. The logarithmic transformation [41] of Equation (8) solved the problem with both the huge values in the binomial coefficients and the small ones related to $\rho^{k}$. It worked well even with the standard Runge-Kutta methods of the second and fourth order [42].

### 2.4. Heuristic Mean-Field Approximation

To capture the characteristics of sparse networks, we may also look for a more sophisticated version of MFA. The one we propose here will be refered to as a heuristic MFA (hMFA) in the remaining part of the paper, since we are not (yet) able to derive all of its ingredients. However, it works quite well in all analyzed cases.

Let us first randomly pick a target and all its closest neighbors. This forms a local configuration. Then, we include $P_{local}$ describing the probability of constructing an unanimous group of influence, for a given configuration:

(12) α+=(1−c)∑i=xkkici(1−c)k−iPlocal,α−=c∑i=xkki(1−c)ick−iPlocal.

In the above formulas, the first part describes initial stage of the process, i.e., probability of obtaining a certain configuration, while $P_{local}$ stands for the probability of constructing an unanimous group of influence. Both $P_{local}$ and *x* are dependent on the variant of the model. In the *no repetition* one they take the following form:

(13) $$P_{local}=\prod_{j=0}^{q-1}\frac{i-j}{k-j},\;\;\;\;x=q.$$

Hence, Equation (12) becomes:

(14) α+=(1−c)∑i=qkkici(1−c)k−i∏j=0q−1i−jk−j,α−=c∑i=qkki(1−c)ick−i∏j=0q−1i−jk−j.

In the *repetition* variant, $P_{local}$ and *x* are:

(15) $$P_{local}={\left(\frac{i}{k}\right)}^{q},\;\;\;\;x=1,$$

and Equation (12) becomes

(16) α+=(1−c)∑i=1kkici(1−c)k−iikq,α−=c∑i=1kki(1−c)ick−icikq.

Now, we can use these $\alpha^{+}$ and $\alpha^{-}$ to compute stationary states, analogously to the ordinary MFA (Equation (6)). Unfortunately, this approach turned out to perform poorly in the case of sparse networks. Thus, it provides only little advantage over the ordinary MFA so far. To change that, we will append some corrections. First, let us introduce a corrected formula for stationary states (see Equation (6)):

(17) $$p^{\prime }=\frac{\alpha }{\alpha -h\beta },$$

where *h* is a positive value, related to the impact of a sparse network on the model’s dynamics. We assume *h* of the form

(18) $$h=1+q/k+2p{\left(q/k\right)}^{2}+32{\left(qk\right)}^{-2}.$$

**Figure 2:**
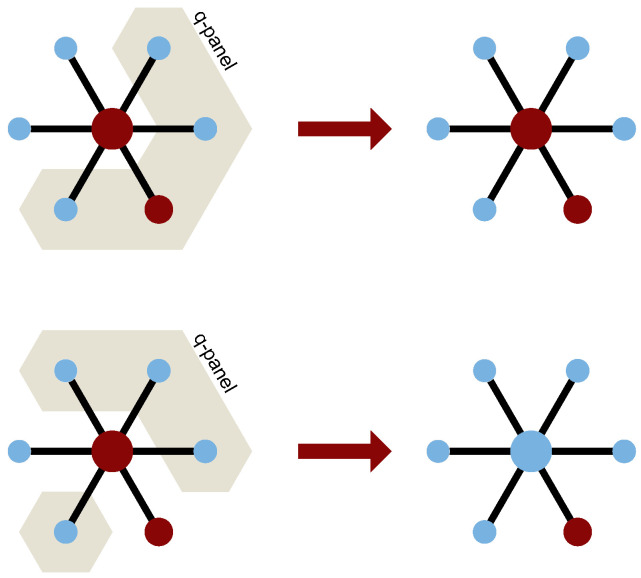
Depiction of two possible configurations. The big circle portrays the target agent, the smaller ones portray its neighbors. Light blue color represents a positive opinion (S=+1), dark red represents a negative one (S=−1). Beige color marks a chosen group of influence. Degree k=6 and size of the influence group q=4 in both cases. The first (top) group of influence is not unanimous and provides no change, while the second one (bottom) is unanimous and leads to change in target’s opinion. The difference is all due to the single neighbor with negative opinion (dark red), being in or outside the group of influence. Probability of containing this neighbor inside the *q*-panel is equal to q/k.

The first element is just 1 and corresponds to the ordinary MFA without any corrections (Equation (6)). The second term, q/k, is there to catch the characteristics of a sparse network. It describes the probability of destroying unanimity within the *q*-panel by a single neighbor (see Figure 2 for a graphical explanation).

Adding the q/k correction has already significantly improved the results yielded by our method. However, the agreement with the ABM simulations was still much worse than that of PA. Thus, we decided to add two other terms based on the calibration of the method to the simulation data. The first of them, $2p{\left(q/k\right)}^{2}$, improves the accuracy of the method in all cases except the low values of *k*. And finally, the $32{\left(qk\right)}^{-2}$ term provides the needed correction for low values of *k*.

Interestingly, the $2p{\left(q/k\right)}^{2}$ term was proposed after calibrating the model with the simulation data in the case k=50, q=5. The last correction was added after the analysis of the k=5 and q=4 case. However, the Formula (18) works reasonably well for all parameter sets we have tested in the preparation phase of this paper. As it will be shown in Section 3.2, the method works only slightly worse than PA, especially in the variant with repetitions, but allows for easier and quicker generation of the results.

### 2.5. Comparison of Methods

In order to compare accuracy of different approximation methods, we introduce the following measure:

(19) $$\Delta =\ln\left[\frac{1}{<p_{s}>}\sum_{i}^{n}\left|p_{s}\left(c_{i}\right)-p_{t}\left(c_{i}\right)\right|:c_{i}\geq 0.6\right],$$

where $p_{s}$ is the simulated value, $p_{t}$ is the value from one of our theoretical models and 0.6 is a cutoff threshold for the simulated values of *c* to remove the finite size effects near the critical value of *p* (see Section 3.1 for explanation). The outcome is normalized by the factor $\frac{1}{<p_{s}>}$, to make comparison between various values of *q* possible. The logarithm function is there to display differences using the same scale in different charts.

## 3. Results

### 3.1. Simulations

**Figure 3:**
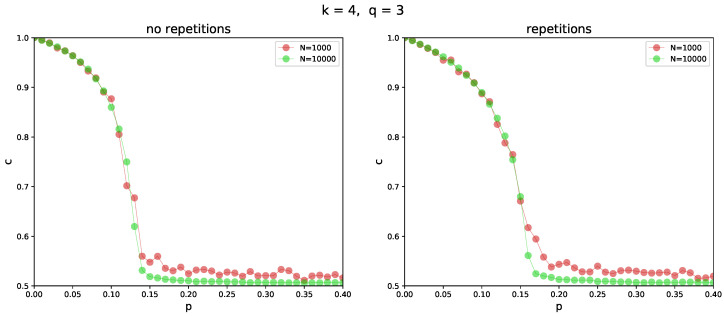
Comparison between the *no repetition* (left) and *repetition* (right) variants in the simulation model (see Section 2.1). Concentration *c* as a function of independence probability *p* is plotted for two different system sizes. Every circle corresponds to the state of the system after 1000 MCS, for a random regular graph with degree k=4, averaged over 100 independent runs. Initial concentration $c_{0}=1$ in all simulations. The size of the group of influence is q=3. Note the longer decay of the ordered phase, characterized by c(t)>0.5, in the case of the smaller system.

We run most of the simulations on random regular graphs of size N=1000, with various degrees *k*. The size of the networks may seem too small at first glance. However, we also checked the simulations on larger networks, and the results were practically the same, except for a small region close to the phase transition (i.e., for c(t) close to 0.5, see Figure 3 for an explanation). In larger systems, the order-disorder phase transition is well defined. In smaller ones, we observe a slower decay of the ordered phase due to the finite size effects [43]. Since a detailed analysis of the phase transition is out of the scope of this paper, we decided to keep the size of the system rather small to reduce computational efforts in the simulations. To ensure that the system reaches its stable state, we set the time horizon T=1000 MCS in each simulation. For each set of the parameters, we perform 100 independent simulations and then average the final concentration, c(T), over these runs.

**Figure 4:**
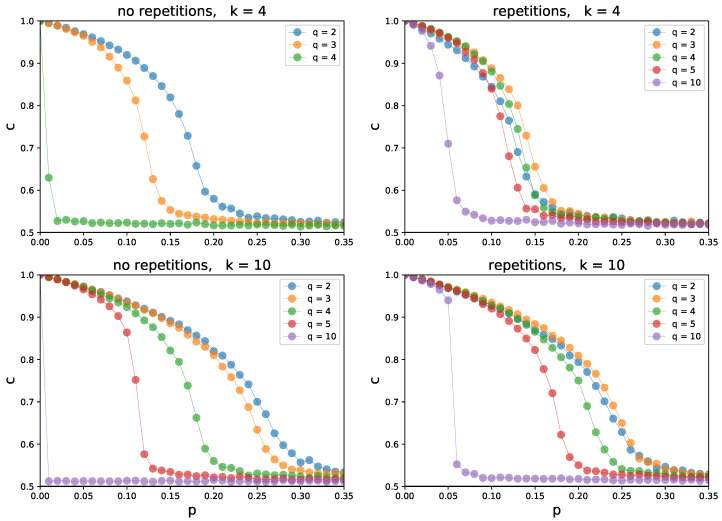
Comparison between the *no repetition* (left) and *repetition* (right) variants in the simulation model (see Section 2.1). Concentration *c* as a function of independence probability *p* is plotted for different sizes *q* of the group of influence. Every circle corresponds to the state of the system after 1000 MCS, for a random regular graph of size N=1000, with degree k=4 (**top**) and k=10 (**bottom**), averaged over 100 independent runs. Initial concentration $c_{0}=1$ in all simulations. Please note that in the *repetition* variant, *q* can be greater than *k*.

The actual comparison between *no repetition* and *repetition* variants is shown in Figure 4. Although at first glance both variants behave similarly, there are some major differences. First of all, in the *no repetition* variant, a group of influence cannot be greater than the degree of a target (q≤k). It is not an issue in the *repetition* one, as we can choose a single neighbor multiple times, when constructing the group of influence. In the *no repetition* variant with q=k, the system becomes disordered (c≈0.5) whenever independence is present (p>0). This is not the case in the *repetition* one. In addition to that, the models behave differently for other values of *q* as well. For larger influence groups, the system in the *no repetition* variant becomes disordered for much smaller probabilities of independence *p* than in the *repetition* one. The closer the value of *q* to *k*, the greater the difference. It is an expected, yet important, outcome. The greater the degree *k* in respect to *q*, the lower the probability of choosing a single neighbor multiple times in the *repetition* variant and thus, the less visible the differences between the variants.

### 3.2. Approximation Methods

The ABMs constitute state-of-the-art tools to simulate complex systems and emergent phenomena, including opinion dynamics on networks. However, understanding and analyzing the ABMs is very challenging. The behavior of those models often depends on many parameters. Even in the case of the simple *q*-voter model, we have to deal with the size of the system *N*, the independence *p*, the degree *k* (which fortunately is the same for all nodes in a random regular graph) and the size *q* of the group of influence. Exploring the parameter space and discovering the impact of different parameter sets on the time evolution of the model can be very time-consuming and usually requires a high-performance computing infrastructure. In many cases, running the model with all possible combinations of parameters is infeasible. On the other hand, varying only one chosen parameter at time may lead to overlooking some interesting patterns in the behavior of the system. This is actually why one is still interested in some analytical approximations of an agent-based system. Although some microscopic details of the dynamics may be ignored, those approximations generate results much faster than computer simulations of ABMs.

As mentioned before, we are going to compare four approximation methods, i.e., MFA, naMFA, hMFA and PA, with the results of our simulations. We are not only interested in the overall agreement with ABMs, but also in the ease of producing the results. Based on the findings for the *q*-voter model so far, we expect that the closer the network is to a complete graph, the more accurate the methods should be. Let us check how well they perform in less obvious cases.

**Figure 5:**
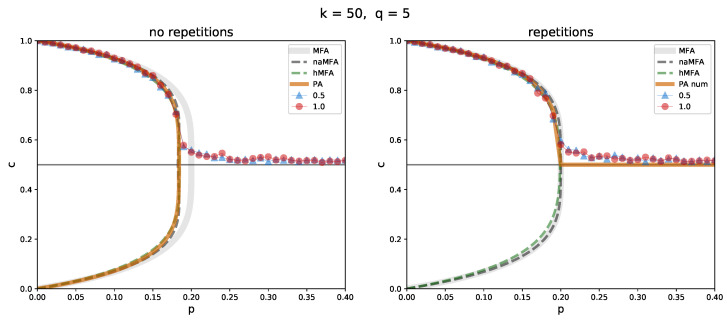
Comparison between the simulation results and the approximation methods in the *no repetition* (**left**) and *repetition* (**right**) variants of the model, for a random regular graph of size N=1000, with the degree k=50 and the size of a group of influence q=5. The solid, light gray line indicates ordinary MFA, the dashed dark gray one indicates naMFA, the dashed green one indicates hMFA, and the solid orange one indicates PA. Simulation results for the initial concentration $c_{0}=0.5$ (blue triangles) and 1 (red circles) are shown. In the *repetition* variant, PA is obtained numerically.

First, a dense network (with the degree k=50) was considered (Figure 5). Although it is still far from a complete graph (for which we would have k=999), it is dense enough for almost all approximation methods to perform well and nearly identically. The only one falling behind is the ordinary mean-field approximation in the *no repetition* variant. The MFA does not utilize the information about the density of the network and assumes the possibility of repetitions when constructing a group of influence. Still, differences between the variants of the model remain small for such a large value of *k*, as mentioned in the Section 2.1.

**Figure 6:**
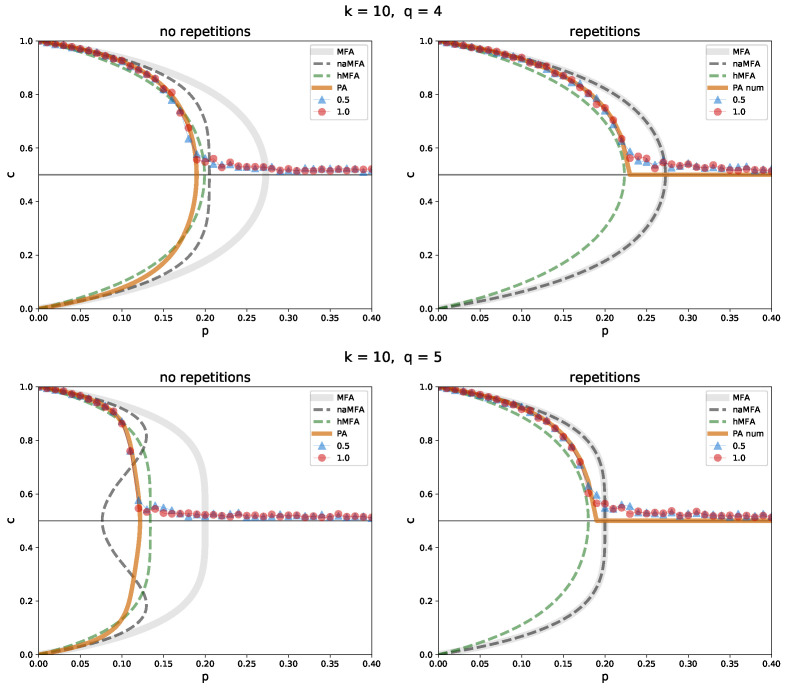
Comparison between the simulation results and the approximation methods in the *no repetition* (**left**) and *repetition* (**right**) variants, for a random regular graph of size N=1000, with the degree k=10 and the sizes of the group of influence q=4 (**top**), and 5 (**bottom**). The solid, light gray line indicates ordinary MFA, the dashed dark gray one indicates naMFA, the dashed green one indicates hMFA, and the solid orange one indicates PA. Simulation results for the initial concentration $c_{0}=0.5$ (blue triangles) and 1 (red circles) are shown. In the *repetition* variant, PA is obtained numerically.

Next, a sparser network (degree k=10) was examined (Figure 6). Here, the differences between the methods and variants become visible. As mentioned earlier, the system in the *no repetition* variant disorders sooner, i.e., for lower values of *p*, than in the *repetition* one. This becomes even more apparent as we increase *q* from 4 to 5. As expected, the ordinary MFA performs poorly in the *repetition* variant and terribly in the *no repetition* one. The network-aware MFA works slightly better in the *no repetition* variant, but only for q=4. For values of *q* approaching *k* it fails as well. Note that the naMFA method in the *repetition* version is equivalent to ordinary MFA. The pair approximation performs best in all cases studied. However, the heuristic MFA is not far behind. Moreover, it has a significant advantage over the PA—it uses a closed-form formula in both variants of the model.

**Figure 7:**
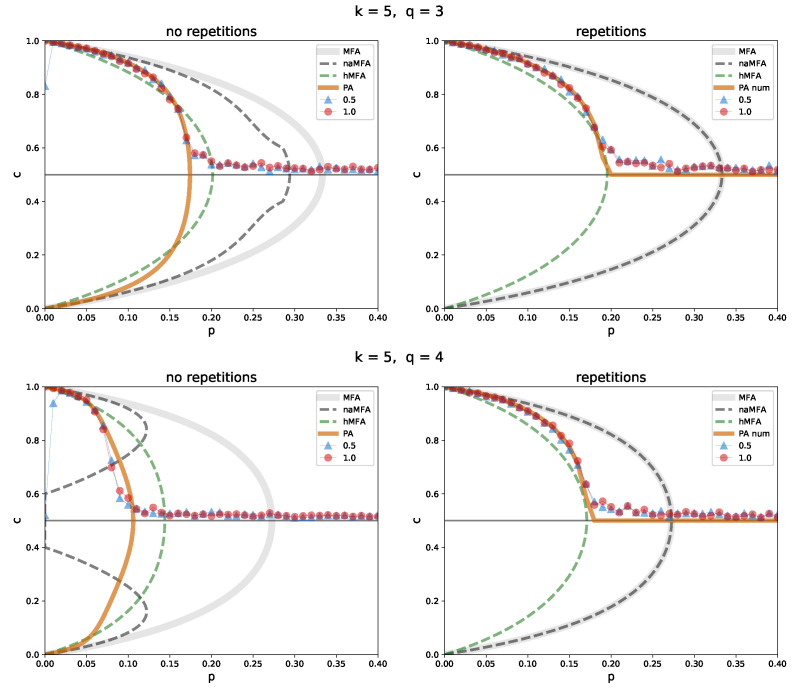
Comparison between the simulation results and the approximation methods in the *no repetition* (**left**) and *repetition* (**right**) variants, for a random regular graph of size N=1000, with the degree k=5 and the sizes of the group of influence q=3 (**top**), and 4 (**bottom**). The solid, light gray line indicates ordinary MFA, the dashed dark gray one indicates naMFA, the dashed green one indicates hMFA, and the solid orange one indicates PA. Simulation results for the initial concentration $c_{0}=0.5$ (blue triangles) and 1 (red circles) are shown. In the *repetition* variant, PA is obtained numerically.

Last but not least, a very sparse network (k=5) was analyzed (Figure 7). Here, inaccuracies of the methods are magnified. Both ordinary MFA and naMFA perform terribly, regardless of the variant of the model or the value of *q*. The PA is still the most accurate method, although the hMFA is again not that far behind. Especially in the *repetition* variant, for which the closed form of the PA cannot be obtained. Since in this case one must rely on a numerical solution of the PA, the hMFA may be an attractive alternative.

**Figure 8:**
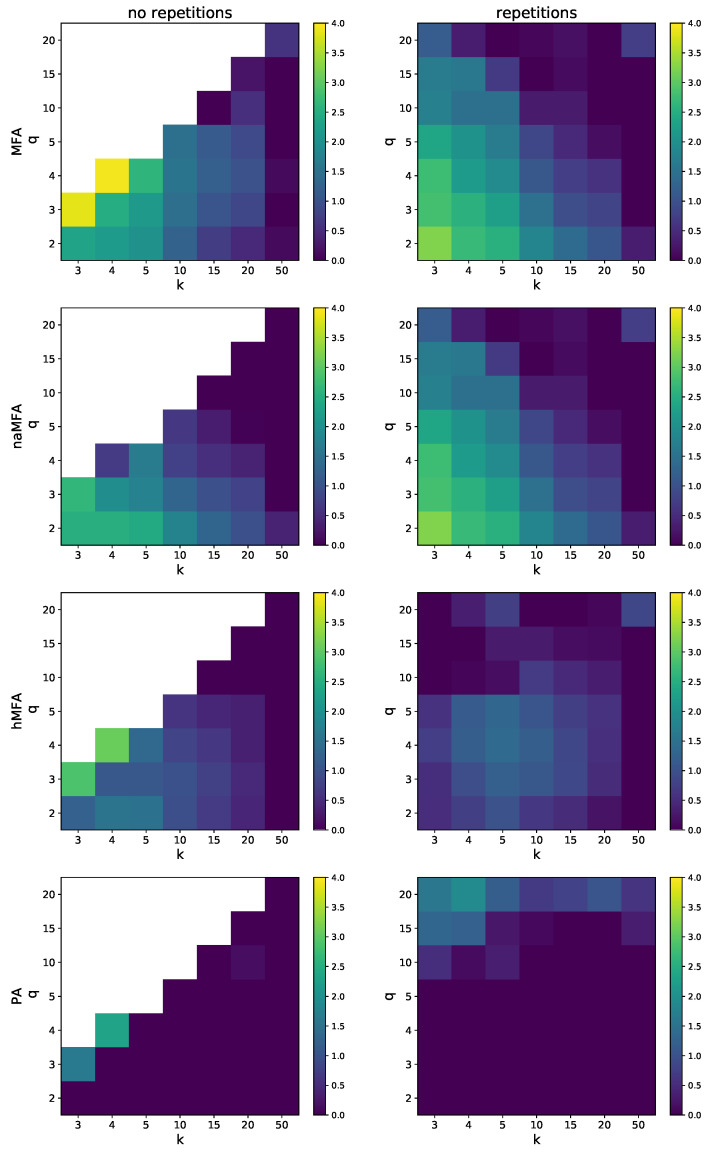
Difference Δ (Equation (19)) between the simulation results and the ordinary MFA (first row), the naMFA (second row), the hMFA (third row), and the PA (fourth row), in the *no repetition* (**left**) and *repetition* (**right**) variants.

The summary of the differences between various approximation methods is presented in Figure 8. The methods are presented from top to bottom in the order of growing accuracy: MFA, naMFA, hMFA and PA. Note that the differences are displayed in a logarithmic scale. The first row corresponds to the ordinary MFA. As already mentioned, it performs satisfactorily only for large values of *k* and fails miserably in all other cases. The second row presents the network aware MFA, a slight modification of MFA with an attempt to capture some aspects of the underlying network. In the *no repetition* variant it performs slightly better. The third row shows the heuristic MFA (hMFA). Overall it yields better accuracy than the former methods, especially in the *repetition* variant. The last row is the PA—the most accurate method, yet it is still not perfect (see k=q=3 and k=q=4 in the *no repetition* variant). Moreover, in the *repetition* variant, its performance becomes worse when *q* is large (see q=20). It is not a numerical artifact, but a property of the PA itself.

## 4. Discussion and Conclusions

This paper consists of two parts. First, we studied the differences between the *repetition* and *no repetition* variants of the *q*-voter model. We found out that differences between them occur and become significant for sparse networks. Thus, one cannot simply use these variants interchangeably without any information.

Secondly, we examined two known approximation methods: the mean-field approximation and the pair approximation, and proposed two additional ones: the network-aware MFA and the heuristic MFA. We compared all these methods in terms of accuracy with the results of agent-based simulations. Two findings stand out as the most significant in our opinion. First, the PA remains the most accurate method overall. Unfortunately, it provides a closed form solution only in the *no repetition* variant. In the *repetition* one we must rely on numerical methods. Second, the hMFA is quite accurate as well. Moreover, it yields formulas much simpler than PA, and thus allows for an analytical solution in both variants of the model. As such, it is an alternative worth considering.

## Data Availability

The data presented in this study are available on request from the corresponding author.
